# Persistent C‐peptide is associated with reduced hypoglycaemia but not HbA_1c_ in adults with longstanding Type 1 diabetes: evidence for lack of intensive treatment in UK clinical practice?

**DOI:** 10.1111/dme.13960

**Published:** 2019-06-27

**Authors:** S. M. Marren, S. Hammersley, T. J. McDonald, B. M. Shields, B. A. Knight, A. Hill, R. Bolt, T. I. Tree, B. O. Roep, A. T. Hattersley, A. G. Jones, R. A. Oram, on behalf of the TIGI consortium

**Affiliations:** ^1^ Institute of Biomedical and Clinical Science, University of Exeter Medical School, Exeter Exeter UK; ^2^ NIHR Exeter Clinical Research Facility University of Exeter Medical School Exeter UK; ^3^ Blood Sciences Royal Devon and Exeter NHS Foundation Trust Exeter UK; ^4^ Department of Immunobiology School of Immunobiology & Microbial Sciences Kings College London London UK; ^5^ NIHR Biomedical Research Centre Guys and St Thomas’ NHS Foundation Trust and Kings College London London UK; ^6^ Department of Diabetes Immunology Diabetes & Metabolism Research Institute at the City of Hope National Medical Center Duarte CA USA; ^7^ Department of Immunohaematology & Blood Transfusion Leiden University Medical Center Leiden The Netherlands

## Abstract

**Aims:**

Most people with Type 1 diabetes have low levels of persistent endogenous insulin production. The Diabetes Control and Complications Trial showed that close to diagnosis preserved endogenous insulin was associated with lower HbA_1c_, hypoglycaemia and complication rates, when intensively treated. We aimed to assess the clinical impact of persistent C‐peptide on rate of hypoglycaemia and HbA_1c_ in those with long duration (> 5 years) Type 1 diabetes.

**Methods:**

We conducted a cross‐sectional case–control study of 221 people (median age 24 years) with Type 1 diabetes. We confirmed ongoing endogenous insulin secretion by measuring C‐peptide after a mixed‐meal tolerance test. We compared self‐reported hypoglycaemia (*n* = 160), HbA_1c_, insulin dose and microvascular complications (*n* = 140) in those with preserved and low C‐peptide.

**Results:**

Stimulated median (IQR) C‐peptide was 114 (43, 273) pmol/l and < 3 (< 3, < 3) pmol/l in those with preserved and low C‐peptide respectively. Participants with preserved C‐peptide had lower reported monthly rates of hypoglycaemia, with 21% fewer symptomatic episodes, 5.9 vs. 7.5 [incidence rate ratio (IRR) 0.79, *P* = 0.001], and 65% fewer asymptomatic episodes, 1.0 vs. 2.9 (IRR 0.35, *P* < 0.001). Those with preserved C‐peptide had a lower insulin dose (0.68 vs. 0.81 units/kg, *P* = 0.01) but similar HbA_1c_ (preserved 69 vs. low 67 mmol/mol, *P* = 0.06).

**Conclusions:**

Adults with Type 1 diabetes and preserved endogenous insulin production receiving usual care in the UK have lower daily insulin doses and fewer self‐reported hypoglycaemic episodes, but no difference in HbA_1c_. This is consistent with non‐intensive treatment in previous studies, and suggests a need to consider therapy intensification to gain full benefit of preserved endogenous insulin.

## What's new?

- Little is known about the clinical impact of persistent C‐peptide in long‐duration Type 1 diabetes.
- We found that in adults with long‐duration Type 1 diabetes, persistent C‐peptide was associated with reduced self‐reported hypoglycaemia and insulin dose but was not associated with reduced HbA_1c_.
- This suggests that individuals with persistent C‐peptide receiving routine care are potentially undertreated.
- Routinely testing C‐peptide and subsequently setting personalized targets for treatment intensification in adults with persistent C‐peptide may improve glycaemic control and complication rates in this group.

**What's new?**

- Little is known about the clinical impact of persistent C‐peptide in long‐duration Type 1 diabetes.
- We found that in adults with long‐duration Type 1 diabetes, persistent C‐peptide was associated with reduced self‐reported hypoglycaemia and insulin dose but was not associated with reduced HbA_1c_.
- This suggests that individuals with persistent C‐peptide receiving routine care are potentially undertreated.
- Routinely testing C‐peptide and subsequently setting personalized targets for treatment intensification in adults with persistent C‐peptide may improve glycaemic control and complication rates in this group.

## Introduction

Recent work has shown that many individuals with long duration Type 1 diabetes continue to produce low levels of endogenous insulin; however, the clinical significance of this is uncertain. β‐Cell function declines with increasing disease duration in Type 1 diabetes and this was assumed to progress to total β‐cell loss 1. Recent studies have demonstrated persistent endogenous insulin in many people with long duration Type 1 diabetes 2, 3, 4, 5, 6. Although there is rapid initial decline post diagnosis, insulin secretion reaches a plateau after ~ 7 years post diagnosis 7. Using highly sensitive C‐peptide assays up to 80% of those with long duration Type 1 diabetes (median duration 18 years) have been shown to have low‐level, detectable endogenous insulin secretion 5. Furthermore, some people with long duration Type 1 diabetes have surprisingly high levels of endogenous insulin, with 8–15% of those diagnosed in adulthood having either a serum C‐peptide > 0.2 nmol/l or a urine C‐peptide creatinine ratio of > 0.2 nmol/mmol 5, 6. The strongest clinical associations of C‐peptide appear to be disease duration and age at diagnosis, with those diagnosed younger being much less likely to have persistent C‐peptide 2, 5, 6, 8.

The Diabetes Control and Complications Trial (DCCT) and islet cell transplant studies have provided evidence for the clinical significance of persistent C‐peptide. Grouped and continuous prospective analyses of DCCT showed higher C‐peptide levels were associated with lower insulin dose, improved glycaemic control, fewer microvascular complications and markedly lower rates of hypoglycaemia 9, 10, 11. These findings were seen only in the intensively treated arm of DCCT, where among intensively treated participants persistent postprandial blood C‐peptide > 200 pmol/l was associated with a reduction in HbA_1c_ and a 65% risk reduction in severe hypoglycaemia when compared with those with C‐peptide < 200 pmol/l 9. Findings from DCCT highlight that the benefit of persistent C‐peptide may arise from allowing tighter glucose control with intensive treatment through protection from hypoglycaemia. Additional data from islet transplant recipients reveal that restoration of even partial β‐cell function improves glycaemic control, variability and hypoglycaemic awareness along with reducing rates of hypoglycaemia 12, 13, 14, 15, 16. The effects of improved β‐cell function in islet transplantation appear continuous and not linked to an absolute threshold, with hypoglycaemic episodes in particular often improving with minimal graft function 16. It is possible that C‐peptide may have direct protective effect on complications 17 although a recent therapeutic trial of C‐peptide did not achieve its primary endpoint 18. These results are important evidence for international efforts to prevent or reverse β‐cell loss 19. Although DCCT provides clear evidence of benefit from preserved endogenous insulin secretion in an intensively treated trial setting, and studies of islet cell transplants show the clear benefit of restoring relatively large amounts of endogenous insulin secretion, the impact of preserved endogenous insulin in people with long‐standing diabetes receiving usual clinical care is unclear.

We aimed to assess the clinical impact of preserved endogenous insulin secretion, measured using C‐peptide, in people with long duration Type 1 diabetes.

## Methods

The TIGI (Type 1 diabetes, Immunology, Genetics and endogenous Insulin production) study is a cross‐sectional, observational case–control study of people with long duration Type 1 diabetes in the UK 7. We recruited participants from the cross‐sectional UNITED (Using pharmacogeNetics to Improve Treatment in Early onset Diabetes) study, a population‐based study of those diagnosed with diabetes before age 30 years (and aged under 50 at recruitment) 5. Potential TIGI participants were selected on the basis of diabetes duration > 5 years and being in either the top or bottom quintile of urinary C‐peptide to creatinine ratio for their diabetes duration in UNITED 5. All participants included in TIGI had clinically defined Type 1 diabetes diagnosed under the age of 30 years, were treated with insulin from diagnosis and lived in the south west of the UK. Those with renal impairment were excluded from the analysis because C‐peptide is not a reliable measure of endogenous insulin production due to its renal excretion 20. Potential participants with a urinary C‐peptide to creatinine ratio > 0.2 nmol/l had glutamic acid decarboxylase (GAD) and islet antigen 2 (IA2) autoantibody testing performed. If autoantibody testing was negative individuals were tested for monogenic diabetes as described previously, and were excluded if found to have monogenic diabetes 21. To avoid the inclusion of young‐onset Type 2 diabetes, those with a urinary C‐peptide to creatinine ratio > 0.2 nmol/l who were islet autoantibody negative were excluded if they had a BMI > 30 kg/m^2^. Some 96% of participants were white British. All participants provided informed consent and the National Research Ethics Service Committee South West approved the study (13/SW/0312).

### Confirmation of C‐peptide status

C‐peptide status was confirmed using a standard mixed‐meal tolerance test. This test was either performed at the Exeter National Institute for Health Research Clinical Research Facility, or at home where participants were visited by the study nurse. All participants fasted from midnight and did not take their morning insulin. Individuals were given a standard mixed‐meal tolerance test (Fortisip Compact, Nutricia, Trowbridge, UK) consisting of 160 ml containing per 100 ml: 240 kcal, 9.6 g protein, 9.3 g fat and 29.7 g carbohydrate. Participants attending the clinical research facility had a full multiple time point mixed‐meal tolerance test, with samples taken at 0, 30, 60, 90 and 120 min post meal. Participants visited at home had an abbreviated single time point mixed‐meal tolerance test, with a blood sample taken at 90 min post meal. Serum C‐peptide was analysed using a direct electrochemiluminescence immunoassay (Roche Diagnostics, Mannheim, Germany). The limit of detection of the assay is 3.3 pmol/l. We confirmed C‐peptide group, preserved (> 20 pmol/l) or low (< 10 pmol/l) using serum C‐peptide post mixed‐meal tolerance test to ensure participants remained in the pre‐defined groups. Eight of the recruited participants were excluded from further analysis as a result of discordant C‐peptide on mixed‐meal tolerance testing. These participants all had high C‐peptide in the UNITED study, but serum C‐peptide < 20 pmol/l after mixed‐meal tolerance test.

### Assessment of hypoglycaemia

Participants completed a modified Clarke's hypoglycaemia questionnaire to assess rate and awareness of hypoglycaemia at the time of mixed‐meal tolerance test. The questionnaire is comprised of eight multiple choice questions, with answers being scored as 0 (aware) or 1 (reduced awareness). The maximum score is 7 and a score > 4 indicates reduced hypoglycaemic awareness 22. Rates of hypoglycaemia were determined by response to questions 5 and 6, they record frequency of hypoglycaemic episodes in the last month (defined as blood glucose < 3.5 mmol/l) with and without symptoms during the episode respectively, as described previously 23. Frequency of episodes was taken as a monthly average; those answering ‘1–3 episodes in the last month’ were averaged to 2, ‘once a week’ to 4 (1 × 4), ‘2–3 episodes per week’ to 10 (2.5 × 4), ‘4–5 episodes per week’ to 18 (4.5 × 4), and ‘almost daily’ to 25.

### Assessment of HbA_1c_ and microvascular complications

HbA_1c_ was measured at the study visit and a historic HbA_1c_ mean calculated from a local laboratory records. With informed consent we collected historic glycaemic control data from a biochemistry laboratory download of all recorded samples over the preceding 12 years in participants from our local area. HbA_1c_ was measured at this time using ion exchange chromatography HPLC on the TOSOH G8 analyser (TOSOH Diagnostics, Tokyo, Japan) and standardized to IFCC. Historic HbA_1c_ mean was calculated for each participant from all available results prior to recruitment [median (IQR) of 18 (12, 26) observations over 8 (5, 10) years].

For participants from our local area**,** clinical data on microvascular complications were obtained from hospital laboratory and retinal screening records. These records were not available for participants whose general practice used the laboratory and retinal screening service of other regional hospitals. Retinopathy status was obtained from the participant's most recent retinal screening record. The worst grade of retinopathy identified at the retinal screening visit prior to recruitment was recorded. Nephropathy status was defined according to whether an individual had ever had clinically defined microalbuminuria, as based on their biochemistry records. Microalbuminuria was defined as having two of three consecutive albumin to creatinine ratios high (> 2.5 mg/mmol for men and > 3.5 mg/mmol for women).

### Statistical analysis

Analysis was carried out using Stata Statistical Software: Release 14 (StataCorp, College Station, TX, USA). Continuous variables were assessed for normality on visual examination of a histogram. Subsequently, differences in all clinical parameters were assessed using the Mann–Whitney *U*‐test. Differences in the severity of retinopathy (characterized as none, background, pre‐proliferative or proliferative) were compared using a non‐parametric trend test. Hypoglycaemia rates were considered to follow a Poisson distribution. Therefore, results are displayed as rates and incidence rate ratios, with confidence intervals also in the Poisson distribution. Statistical significance was defined as *P* < 0.05 for all statistical tests. The study recruited people who were in the top or bottom 20th centile of stimulated C‐peptide adjusted for duration. This led to a large difference in C‐peptide between the two pre‐defined groups and we therefore performed comparative analyses of these groups.

## Results

Some 70 participants with preserved C‐peptide and 151 with low C‐peptide were included in this analysis (characteristics presented in Table 1). The majority were adults, the median (IQR) age 24 (17, 39) years. Median (IQR) C‐peptide was 114 (43, 273) pmol/l in the preserved C‐peptide group, and < 3 (< 3, < 3) pmol/l in the low C‐peptide group. Although duration of diabetes was similar between the two groups (median 13 years in both groups, *P* = 0.2), the preserved C‐peptide group were diagnosed at an older age, 15 vs. 6 years (*P* < 0.0001).

**Table 1:** Cohort characteristics

Characteristics	Low C‐peptide	Preserved C‐peptide	*P*‐value
No. of participants	151	70	–
No. male (%)	86 (57)	29 (41)	0.03*
Age at diagnosis (years)	6.1 (3.0, 12.5)	15.1 (12.2, 22.0)	< 0.0001
Age at recruitment (years)	19.9 (14.3, 36.5)	30.9 (20.8, 42.1)	0.0001
BMI (kg/m²)	23.3 (20.2, 26.5)	25.2 (23.3, 27.3)	0.0006
BMI standard deviation score	0.8 (0.1, 1.5)	1.0 (0.5, 1.6)	0.2
Duration of diabetes (years)	13.3 (8.5, 24.5)	12.6 (7.5, 22.0)	0.2
C‐peptide (pmol/l)	<3 (<3, <3)	114 (43, 273)	< 0.0001
HbA_1c_ (mmol/mol)	67 (58, 76)	69 (62, 81)	0.6
HbA_1c_ (%)	8.3 (7.5, 9.1)	8.5 (7.8, 9.6)	0.6
Insulin dose (U/kg in 24 h)	0.81 (0.67, 0.95)	0.68 (0.54, 0.94)	0.01
No. in hypoglycaemia rate analysis (%)	121 (80)	39 (56)	–
No. in historic HbA_1c_ analysis (%)	86 (57)	67 (96)	–

Values are reported as *n* (%) or median (IQR). *P‐*values are Mann–Whitney *U* unless stated otherwise stated; *χ^2^.

### Adults with preserved C‐peptide had lower rates of hypoglycaemia

**Figure 1:**
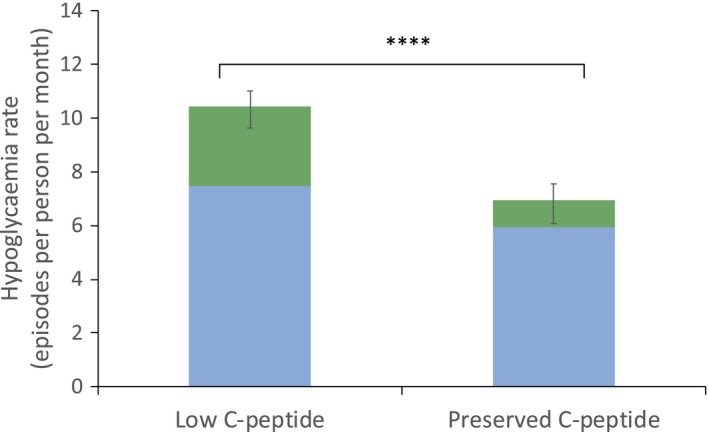
Total monthly rate of hypoglycaemia by C‐peptide group. Rates of aware (blue) and unaware (green) episodes with blood glucose < 3.5 mmol/l per month; derived from Clarke's hypoglycaemia questionnaire questions 5 and 6 respectively. ****P < 0.0001, for low (*n* = 118) vs. preserved (*n* = 39) C‐peptide. Error bars represent 95% confidence intervals.

Hypoglycaemia questionnaires were filled out by 160 participants (Table S1), median (IQR) age 21 (15, 38) years. Some 151 participants completed all parts of questionnaire and we were able to calculate both hypoglycaemia rates and awareness; 9 of 160 partially completed the questionnaire, enabling calculation of hypoglycaemia rates but not hypoglycaemic awareness. The preserved C‐peptide group had 21% fewer symptomatic episodes per month [incidence rate ratio (IRR) 0.79, 95% confidence interval (CI) 0.68–0.91, *P* = 0.001], 5.9 vs. 7.5 episodes/month; and 65% fewer asymptomatic episodes per month (IRR 0.35, 95% CI 0.25–0.48, *P* < 0.001), 1.0 vs. 2.9 episodes/month (Figs 1 and S1). There was no difference in Clarke score between the two groups (*P* = 0.3), or proportion with a reduced hypoglycaemic awareness (score > 4 of 7, 8% vs. 12%, *P* = 0.5; Fig. S2).

### Both the study visit HbA_1c_ and historic HbA_1c_ means were similar in both groups

**Figure 2:**
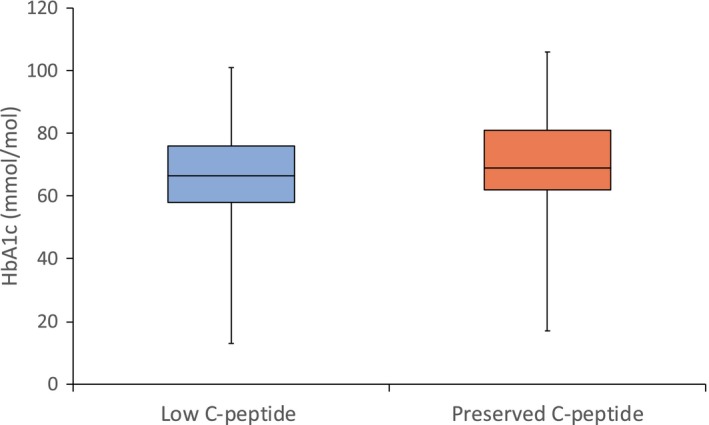
Boxplot of study visit HbA_1c_ by C‐peptide group. Study visit HbA_1c_ was similar in the low (*n* = 148) and preserved (*n* = 70) C‐peptide groups *P* = 0.06. Outliers are shown in Fig. S3.

Participants in the preserved group had a marginal trend towards higher study visit HbA_1c_, 69 vs. 67mmol/mol (8.5% vs. 8.3%), *P* = 0.06 (Figs 2 and S3). Historic HbA_1c_ mean was calculated in 153 participants (Table 1). The historic HbA_1c_ mean was also similar in both groups, 71 vs. 68mmol/mol (8.6% vs. 8.4%) in those with high and low C‐peptide respectively, *P* = 0.4 (Fig. S4).

### Insulin dose was substantially lower in those with preserved C‐peptide production

**Figure 3:**
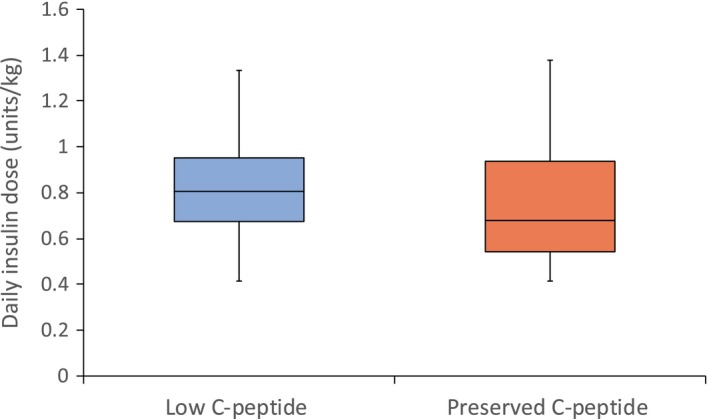
Boxplot of daily insulin dose by C‐peptide group. Insulin dose was lower in the preserved C‐peptide group (*n* = 70) vs. the low (*n* = 151) group, *P* = 0.01. Outliers are shown in Fig. S5.

Participants with preserved C‐peptide received a total daily dose of 0.68 (0.54, 0.94) units/kg, whereas those with low C‐peptide received 0.81 (0.67, 0.95) units/kg, for comparison *P* = 0.01 (Figs 3 and S5).

### Preservation of endogenous insulin secretion was not associated with differences in retinopathy or microalbuminuria

Retinopathy results were available on 130 participants (Table S2). Presence of retinopathy in the preserved C‐peptide group was similar to the low group, 66% vs. 74%, *P* = 0.3 (Fig. S6). There was no difference in grades of retinopathy between the two groups (*P* = 0.5).

The prevalence of microalbuminuria was also similar in participants with high and low C‐peptide. Records were available on 120 participants (Table S2). In these participants 20% and 23% of those with high and low C‐peptide met study criteria for microalbuminuria (*P* = 0.6) (Fig. S6).

### Sensitivity analysis excluding people with C‐peptide > 200 pmol/l

To test whether the effects seen were due purely to those with C‐peptide > 200 pmol/l we performed analysis with these people excluded. The detectable (but < 200 pmol/l) C‐peptide group had 20% fewer symptomatic episodes per month (IRR 0.80, 95% CI 0.68–0.84, *P* = 0.005) and 61% fewer asymptomatic episodes per month (IRR 0.39, 95% CI 0.28–0.55, *P* < 0.001). HbA_1c_ was similar between the two groups (*P* = 0.1), as was daily insulin dose (*P* = 0.1). Rates of retinopathy and microalbuminuria were similar in both groups (*P* = 0.9 and 0.4 respectively). Grade of retinopathy was also similar in both groups (*P* = 0.6).

## Discussion

Our study showed that in adults, low levels of preserved C‐peptide production in long duration Type 1 diabetes in UK clinical practice were associated with reduced self‐reported hypoglycaemia without improvement in HbA_1c_. Rates of both symptomatic and asymptomatic hypoglycaemia were reduced in the preserved C‐peptide group, with similar hypoglycaemic awareness and without differences in either single measure or historic HbA_1c_ mean. However, those with preserved C‐peptide were treated with a lower exogenous insulin dose. Consistent with a lack of difference in HbA_1c_, levels of retinopathy and microalbuminuria were not different when examined in a subset of our study population.

These findings mirror the conventionally treated arm of the DCCT. This suggests a lack of intensive treatment in UK practice and highlights the challenges in achieving tight control outside the closely monitored clinical trial setting. DCCT showed that where intensive diabetes treatment is given, higher levels of C‐peptide are associated with markedly lower hypoglycaemia, HbA_1c_ and microvascular complications even with low levels of secretion (< 200 pmol/l). However, benefits were much less marked where conventional therapy was given 9, 10, 11. Although this may be explained in part by more rapid loss of endogenous insulin secretion in non‐intensively treated participants 11, it may also relate to reductions in hypoglycaemia risk associated with preserved C‐peptide allowing intensification of treatment to a tighter level of glycaemic control. The limitation of achieving optimal glycaemic control with intensive treatment in Type 1 diabetes is usually hypoglycaemia, which prevents up‐titration of insulin doses. The reduced glucose variability and better hypoglycaemia counter‐regulation associated with preservation of endogenous insulin secretion 16, 23, 24, 25 means that with intensive treatment an adult with retained endogenous insulin secretion can obtain a lower HbA_1c_ at an acceptable level of hypoglycaemia than would be possible where endogenous insulin is absent.

Our findings are consistent with previous research on the clinical impact of C‐peptide. Hope *et al*. 23 observed an approximate doubling in self‐reported hypoglycaemia in those with Type 1 diabetes with C‐peptide < 200 pmol/l compared with > 200 pmol/l. This study focused on people who were older when diagnosed, with group durations of 25 vs. 10 years respectively 23. Kuhtreiber *et al*. 8 also used the Clarke score to assess hypoglycaemia. They categorized individuals as having mild, moderate or severe hypoglycaemia, showing that more severe hypoglycaemia was associated with lower levels of C‐peptide. In addition, they found that higher C‐peptide was associated with better glycaemic control and fewer complications. Their study had the benefit of a larger sample size and was thus better powered to assess a difference in complication rates 8. Our data are also aligned with studies of islet cell transplant recipients. In this setting, even minimal graft function, measured by C‐peptide or β score, correlates with reduced hypoglycaemia risk and improved glycaemic variability 12, 13, 14, 15, 16. Vantyghem *et al*. 13 showed that in islet cell transplant recipients partial β‐cell function reduced rates of hypoglycaemia however improvements in glycaemic control and variability required significantly better graft function. Combined these findings point toward endogenous insulin playing a key role in preventing hypoglycaemia; perhaps directly by stopping secretion when blood glucose levels fall or indirectly through counter‐regulatory hormones such as glucagon.

Strengths of our study include that we were able to utilize a highly sensitive C‐peptide assay, allowing for identification and classification of C‐peptide status at historically undetectable levels. We also robustly excluded individuals with both Type 2 and monogenic diabetes, ensuring that those with a high C‐peptide truly had Type 1 diabetes. Additionally, our recruitment process allowed the disease duration of both groups to be the same, removing a potential key confounder from this analysis. Our cohort were not part of a clinical intervention trial and received routine clinical care, making them reflective of current Type 1 diabetes management in the UK, both strengthening our findings and making them relevant to routine care in the UK.

Our study was limited by self‐reported hypoglycaemia and region‐restricted complication data reducing the power to assess a difference in complications. We used a validated hypoglycaemia questionnaire; however, this relied on participants both correctly identifying and recording hypoglycaemic episodes. It is likely that our data underestimate the frequency of hypoglycaemia, due to reduced hypoglycaemic awareness and nocturnal hypoglycaemia. Accurate identification of hypoglycaemia relies on frequent blood glucose self‐monitoring or continuous glucose monitoring, neither of which were part of the study protocol. It would be valuable to carry out continuous glucose monitoring on a cohort of similar participants, looking to remove participant bias and potentially validate our findings. A further limitation of our hypoglycaemia analysis is that participants with low C‐peptide were younger (18.4 vs. 29.3 years), differences in care, and frequency of hypoglycaemia in the paediatric population may potentially influence our findings. Despite studying 69 cases aged under 18 years overall (median age 14 years), with 62 cases in the hypoglycaemia rate analysis, the number of people under 18 years with preserved C‐peptide was very low (6 of 69 and 4 of 62). Because of the absence of young children in the preserved C‐peptide group, our suggestion of potentially intensifying treatment in those with preserved C‐peptide is not applicable to children. This additionally raises the question of whether the high hypoglycaemia rate in young children is related directly to this lack of preserved C‐peptide in those diagnosed young, or to a mixture of metabolic and behavioural factors. The differences in age of study participants with and without preserved C‐peptide could potentially bias our findings of different insulin doses in those with low and high C‐peptide: many (51 of 151) of our low C‐peptide participants were in the pubertal age range (10–16 years), which can affect insulin requirements. However, when we excluded those between the ages of 10 and 16 years, the differences were similar: median daily insulin dose was 0.67 units/kg in the preserved group compared with 0.75 units/kg in the low group (*n* = 166, *P* = 0.1). A further limitation is that we were only able to obtain data on complications on participants based in the Exeter area, due to the availability of medical records. This reduced our power to identify differing rates of retinopathy and microalbuminuria, so we could not rule out smaller differences that may still be clinically relevant. The sample size for our complication analysis provided 80% power (α = 0.05), to detect a difference in proportions of 30% for both retinopathy and microalbuminuria, therefore meaningful differences in complications may not be detected with our limited sample size. A further limiting factor to this analysis was the selection criteria for our study, which excluded people with renal impairment, as C‐peptide is renally excreted and therefore less reflective of endogenous beta cell function in those with impaired renal function 20. Our study assessed endogenous insulin by measuring serum C‐peptide, and is therefore unable to differentiate between an association with preserved endogenous insulin, or a direct association with C‐peptide rather than endogenous insulin.

Notwithstanding these limitations, our study suggests it may be possible to intensify treatment in adults with persistent C‐peptide. DCCT demonstrated that the benefits of persistent C‐peptide production could only be fully utilized when individuals received intensive therapy. Our study focused on those receiving routine clinical care and did not show the improvement of HbA_1c_ associated with persistent C‐peptide in DCCT, with the benefit of maintained endogenous insulin secretion limited to lower insulin dose and less hypoglycaemia. A lack of impact on microvascular complications is therefore unsurprising considering >10 years of HbA_1c_ records showed similar glycaemic control in both groups. We consider the most likely explanation for this finding to be a lack of intensive treatment, showing that factors other than hypoglycaemia limit achievement of HbA_1c_ targets. Clinicians do not routinely test C‐peptide and there are currently no guidelines to treat those with persistent C‐peptide, who are protected from hypoglycaemia, more intensively. Therefore, these people may be considered to have acceptable glycaemic control in practice, when they would be able to achieve tighter glycaemia control and reduce risk of long‐term complications with more intensive treatment, without unacceptable hypoglycaemia. Cautious targeted intensification of treatment in adults with preserved C‐peptide could therefore be a potential clinical strategy to improve control and an important area for future study.

Our study highlights the association of persistent high C‐peptide with reduced hypoglycaemia in adults. Additionally, it demonstrates that higher C‐peptide does not always robustly associate with improved glycaemic control and reduced complications rates. This may be a consequence of all adults being treated to the same glycaemic targets, irrespective of C‐peptide production. The apparent under‐treatment of those with preserved C‐peptide production makes our assessment of any complication benefit difficult.

## Funding sources

This study was funded by the JDRF (strategic research agreement 201301394). SH, BK, AH and RB are core members of the National Institute for Health Research Exeter Clinical Research Facility. ATH and BMS are supported by the NIHR Exeter Clinical Research Facility. BMS is supported as part of the MRC MASTERMIND consortium. TJM is funded by an NIHR clinical senior lecturer fellowship. ATH is supported by a Wellcome Trust Senior Investigator Award (WT098395/Z/12/Z) and an NIHR Senior Investigator award. AGJ is supported by an NIHR Clinician Scientist award. RAO is supported by a Diabetes UK Harry Keen Fellowship. This article presents independent research supported by JDRF, Diabetes UK, the Wellcome Trust and the NIHR Exeter Clinical Research Facility.

The views expressed are those of the authors and not necessarily of JDRF, Diabetes UK, the Wellcome Trust, the National Health Service, the National Institute for Health Research, or the Department of Health.

## Competing interests

None declared.

## Author contributions

RAO, AGJ, TJM and ATH conceived of and designed the study. TT and BR contributed to design of the study. AH and RB recruited participants for the study. SMM, SH, BK, AH and RB were involved in acquisition of data and clinical information. SMM, BMS, AGJ, ATH and RAO contributed to the data analysis. SMM, AGJ and RAO wrote the manuscript which was reviewed by all authors. All authors approved the final version of the manuscript.

## Supporting information


**Figure S1.** Hypoglycaemia rates by C‐peptide group.
**Figure S2.** Reduced hypoglycaemic awareness by C‐peptide group.
**Figure S3.** Boxplot of study visit HbA_1c_ by C‐peptide group.
**Figure S4.** Historic HbA_1c_ mean analysis.
**Figure S5.** Boxplot of daily insulin dose by C‐peptide group.
**Figure S6.** Rates of retinopathy and microalbuminuria by C‐peptide group.
**Table S1.** Hypoglycaemia analysis cohort characteristics.
**Table S2.** Complication analysis cohort characteristics.
