# Supplementary material for: Persistent C‐peptide is associated with reduced hypoglycaemia but not HbA1c in adults with longstanding Type 1 diabetes: evidence for lack of intensive treatment in UK clinical practice?
Source: Diabet Med. 2019 Jun 27;36(9):1092–9. doi: 10.1111/dme.13960 (PMC6790586; doi:10.1111/dme.13960)

Table S1 – Hypoglycaemia analysis cohort characteristics. Values reported as n (%) or median (IQR). *p* values are Mann Whitney U unless otherwise stated, * denotes Chi^2.

Characteristics	Low C-peptide	Preserved C-peptide	*p* value
**No. of participants**	121	39	-
**No. Male (%)**	69 (57)	17 (44)	0.1*
**Age at diagnosis (years)**	5.7 (2.9, 10.8)	15.0 (12.2, 24.0)	<0.0001
**Age at recruitment (years)**	18.4 (13.5, 34.5)	29.3 (20.4, 43.0)	0.0004
**BMI (kg/m²)**	23.3 (19.6, 26.2)	24.5 (22.1, 27.4)	0.02
**BMI standard deviation score**	0.9 (0.1, 1.4)	0.8 (0.3, 1.5)	0.8
**Duration of diabetes (years)**	12.6 (8.3, 20.8)	12.1 (7.5, 21.4)	0.4
**C-peptide (pmol/l)**	<3 (<3, <3)	108 (37, 208)	<0.0001
**HbA1c (mmol/mol)**	67 (57, 75)	68 (59, 80)	0.4
**HbA1c (%)**	8.3 (7.4, 9.0)	8.4 (7.5, 9.5)	0.4
**Insulin dose in 24 h (U/kg)**	0.80 (0.67, 0.95)	0.71 (0.55, 0.94)	0.09
**No. in hypoglycaemia awareness analysis**	113	38	-

Fig. S1 – Hypoglycaemia rates by C-peptide group. Number of hypoglycaemic (blood glucose <3.5mmol/l) episodes per month stratified by aware and unaware episodes. Rates derived from Clarkes questions 5 and 6. Low C-peptide group in blue (aware n=120, unaware n=119) and preserved C-peptide group in orange (n=39). ***p≤0.001 for low vs preserved C-peptide. Error bars represent 95% CI.

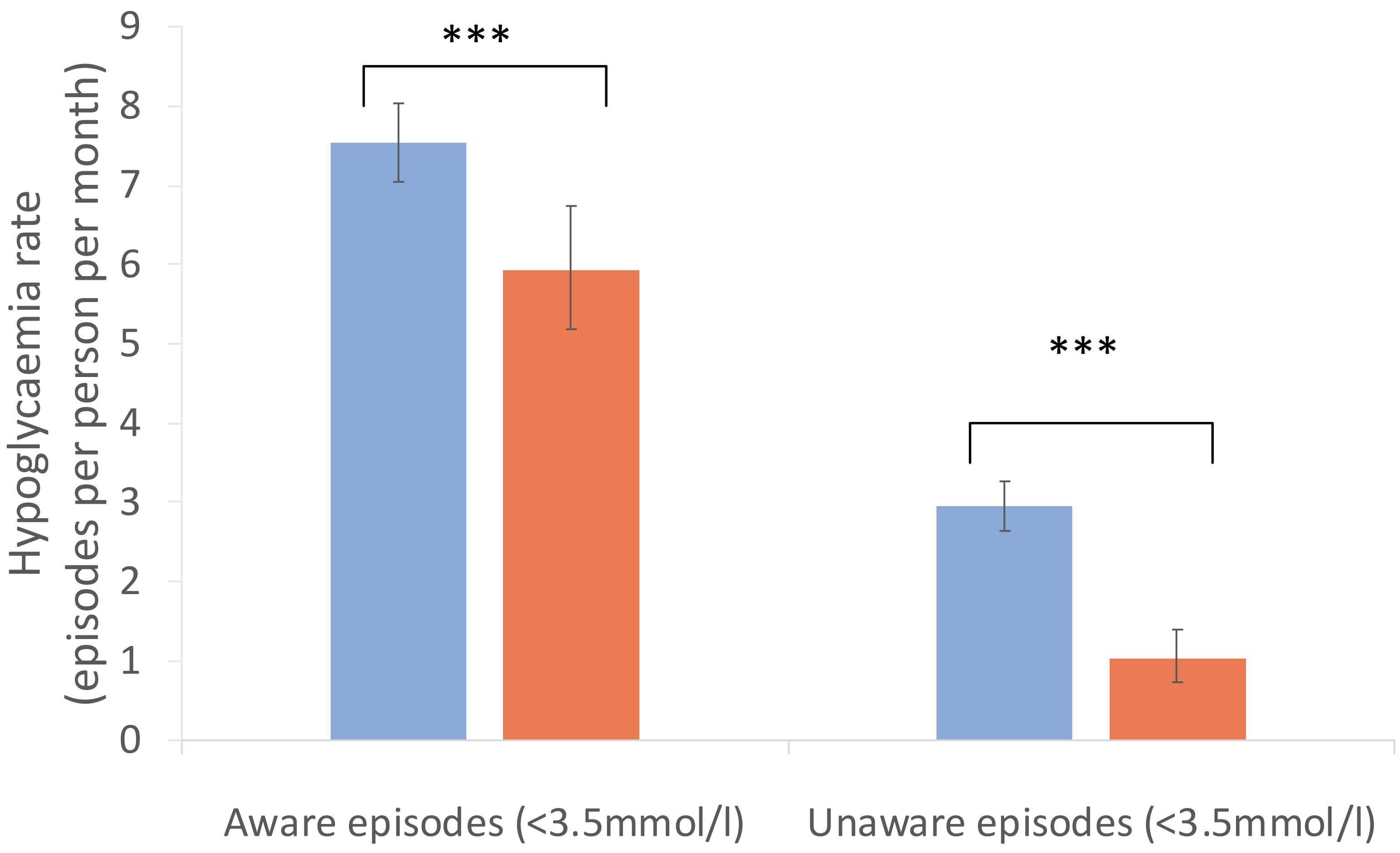

Fig. S2 – Reduced hypoglycaemic awareness by C-peptide group. Proportion of the low (blue, n=113) or preserved (orange, n=38) C-peptide group with reduced hypoglycaemic awareness, defined as a Clarke score >4/7. Hypoglycaemic awareness was similar in both groups, p=0.5.

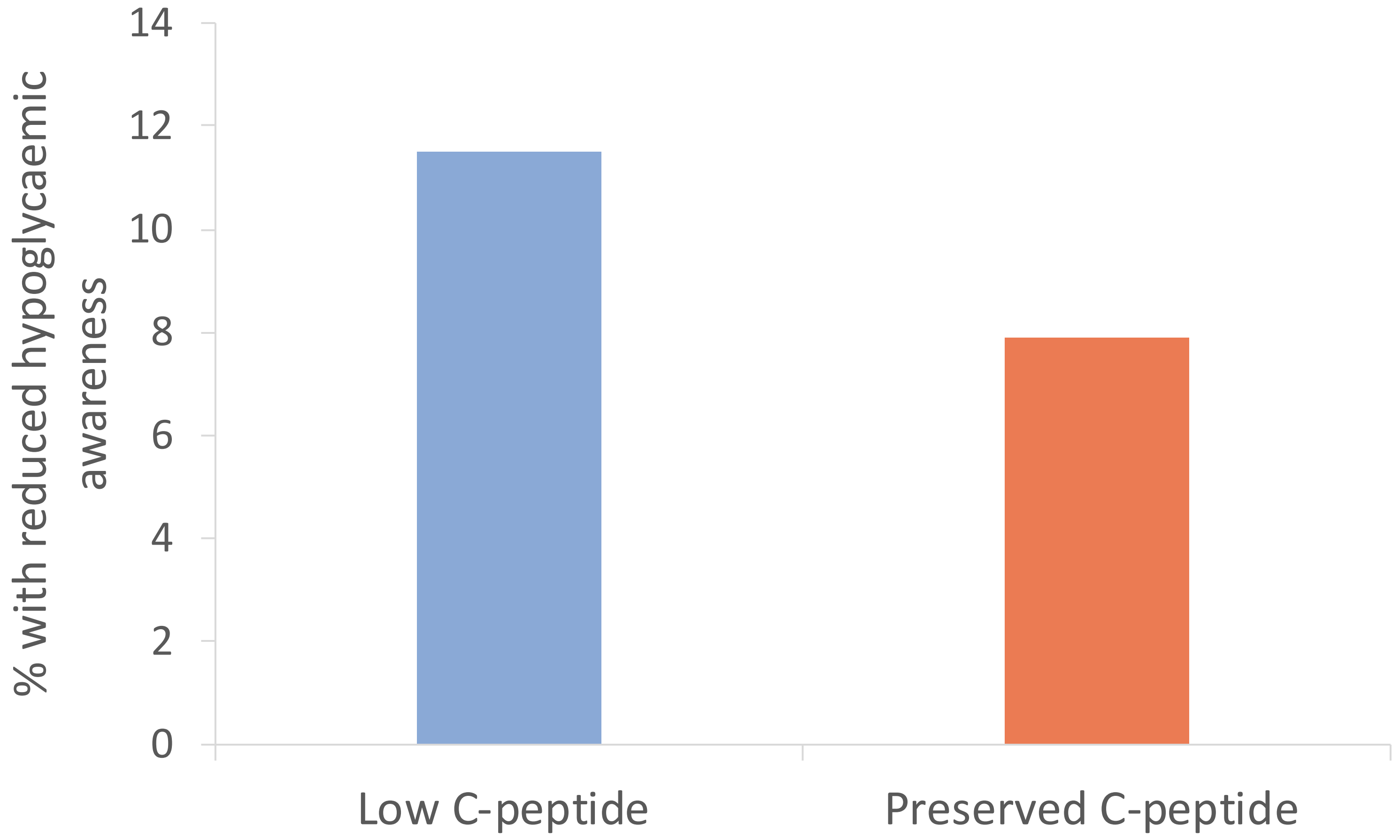

Fig. S3 – Boxplot of study visit HbA1c by C-peptide group. Study visit HbA1c was similar in the low (n=148) and preserved (n=70) C-peptide groups p=0.06. Including outliers.

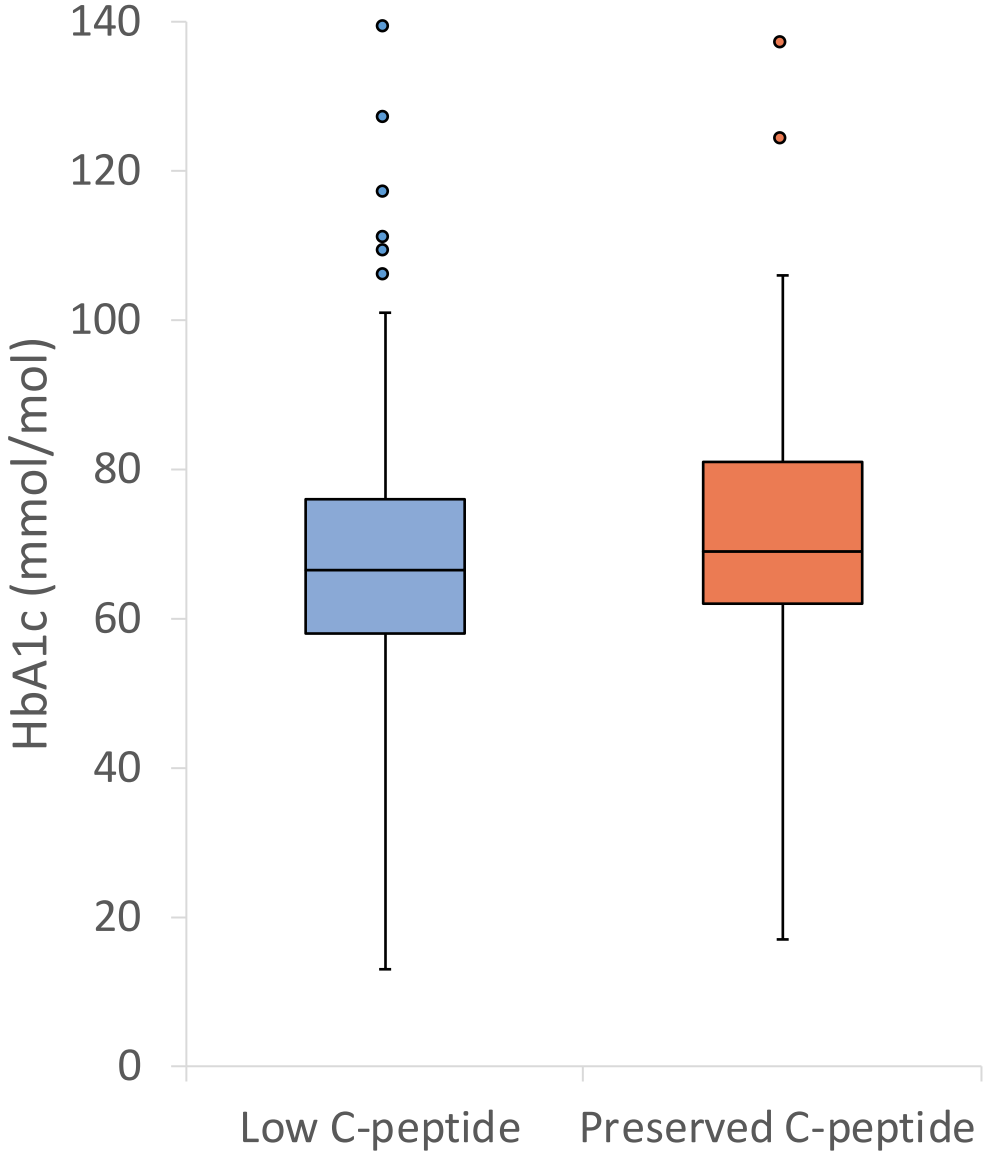

Fig. S4 - Historic HbA1c mean analysis **(a)** Boxplot showing similar historic HbA1c mean in low (n=86) vs preserved (n=67) C-peptide group, p=0.4. **(b)** Scatter graph of study visit HbA1c and historic HbA1c mean (n=153). Spearman's rho = 0.72, p<0.0001. Low C-peptide group (n=86) shown in blue and preserved C-peptide group (n=67) in orange. **(c)** Boxplot showing similar study visit HbA1c (n=153) vs historic HbA1c mean (n=153), p=0.1.

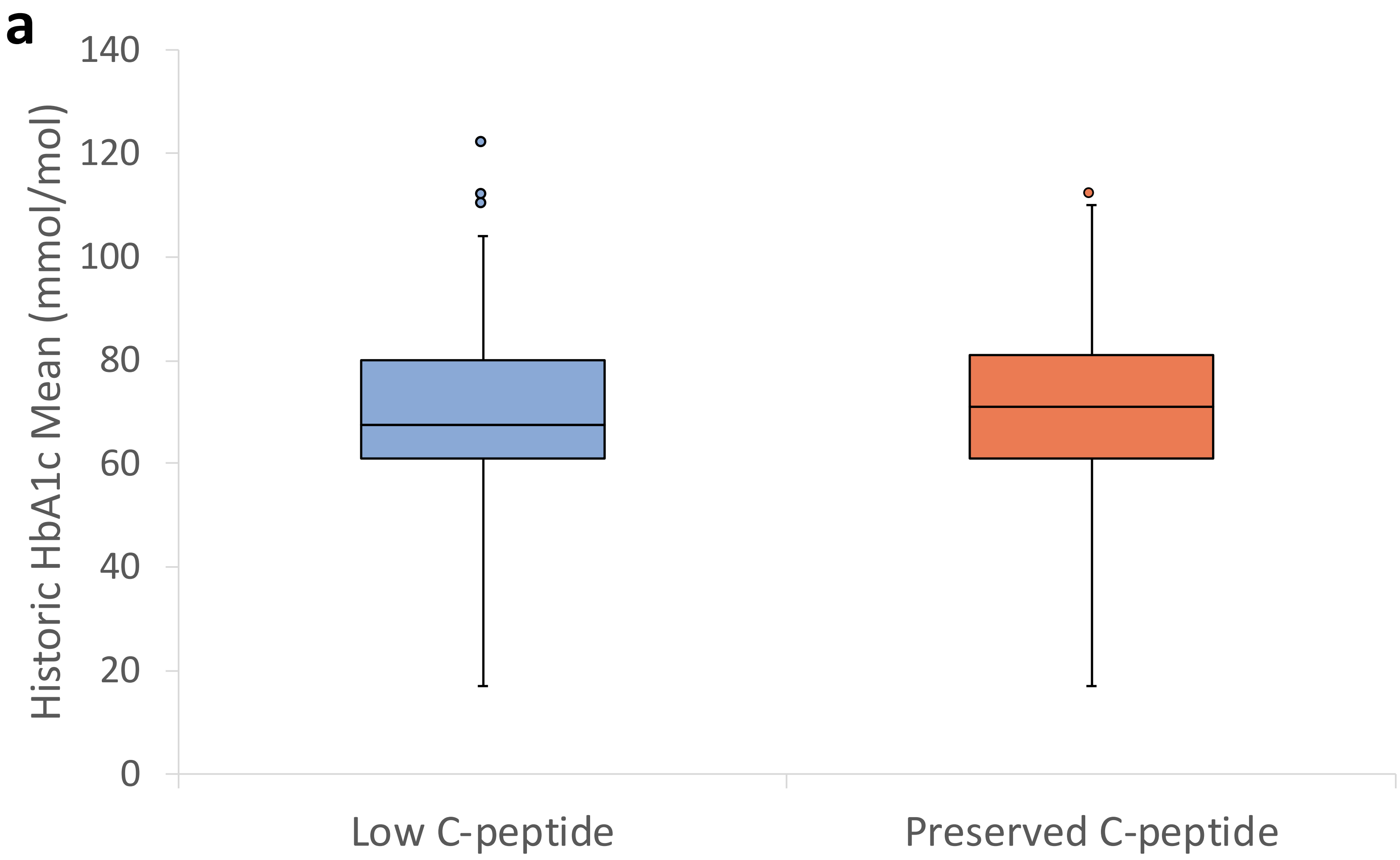

Fig. S4 - Historic HbA1c mean analysis **(a)** Boxplot showing similar historic HbA1c mean in low (n=86) vs preserved (n=67) C-peptide group, p=0.4. **(b)** Scatter graph of study visit HbA1c and historic HbA1c mean (n=153). Spearman's rho = 0.72, p<0.0001. Low C-peptide group (n=86) shown in blue and preserved C-peptide group (n=67) in orange. **(c)** Boxplot showing similar study visit HbA1c (n=153) vs historic HbA1c mean (n=153), p=0.1.

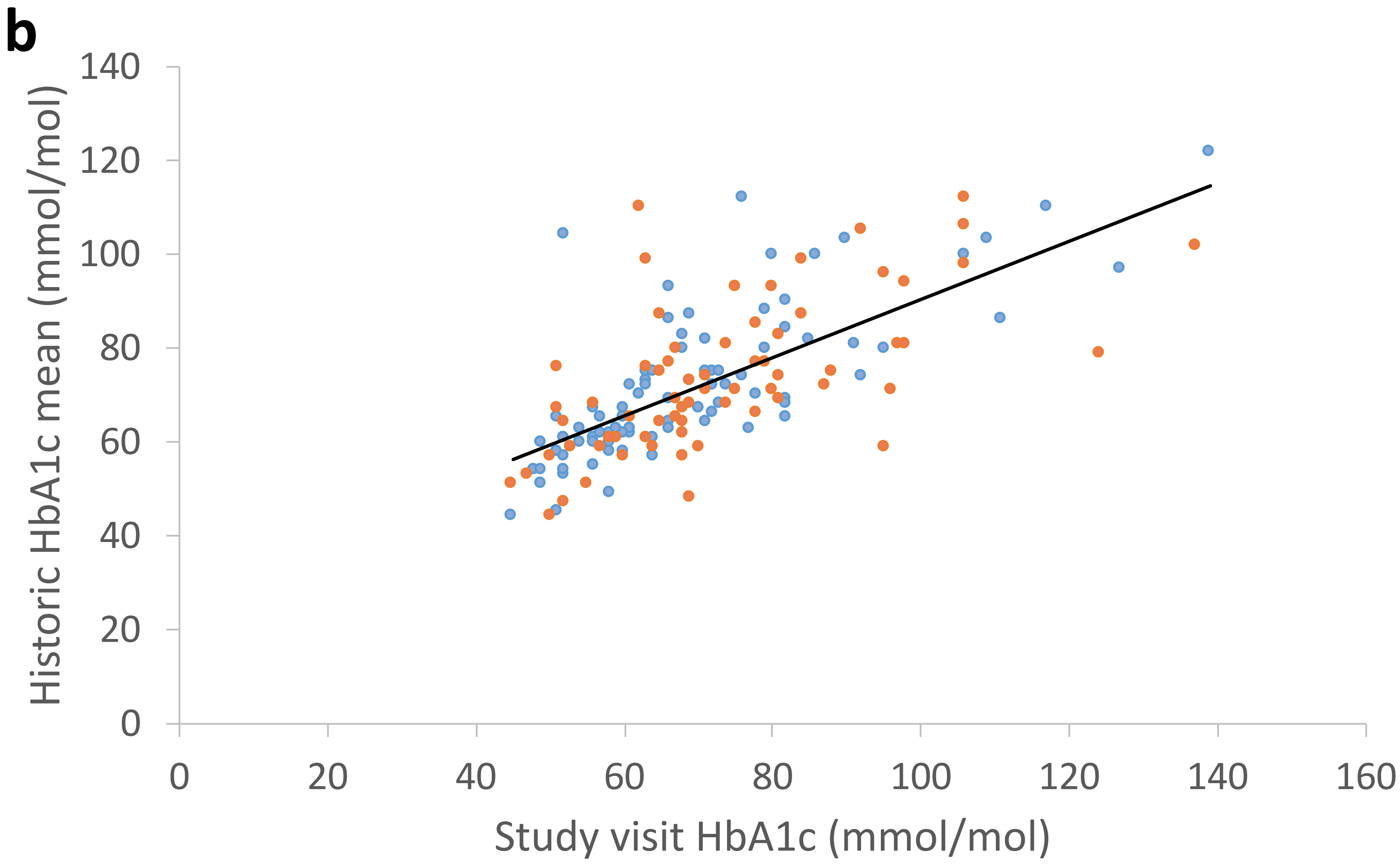

Fig. S4 - Historic HbA1c mean analysis **(a)** Boxplot showing similar historic HbA1c mean in low (n=86) vs preserved (n=67) C-peptide group, p=0.4. **(b)** Scatter graph of study visit HbA1c and historic HbA1c mean (n=153). Spearman's rho = 0.72, p<0.0001. Low C-peptide group (n=86) shown in blue and preserved C-peptide group (n=67) in orange. **(c)** Boxplot showing similar study visit HbA1c (n=153) vs historic HbA1c mean (n=153), p=0.1.

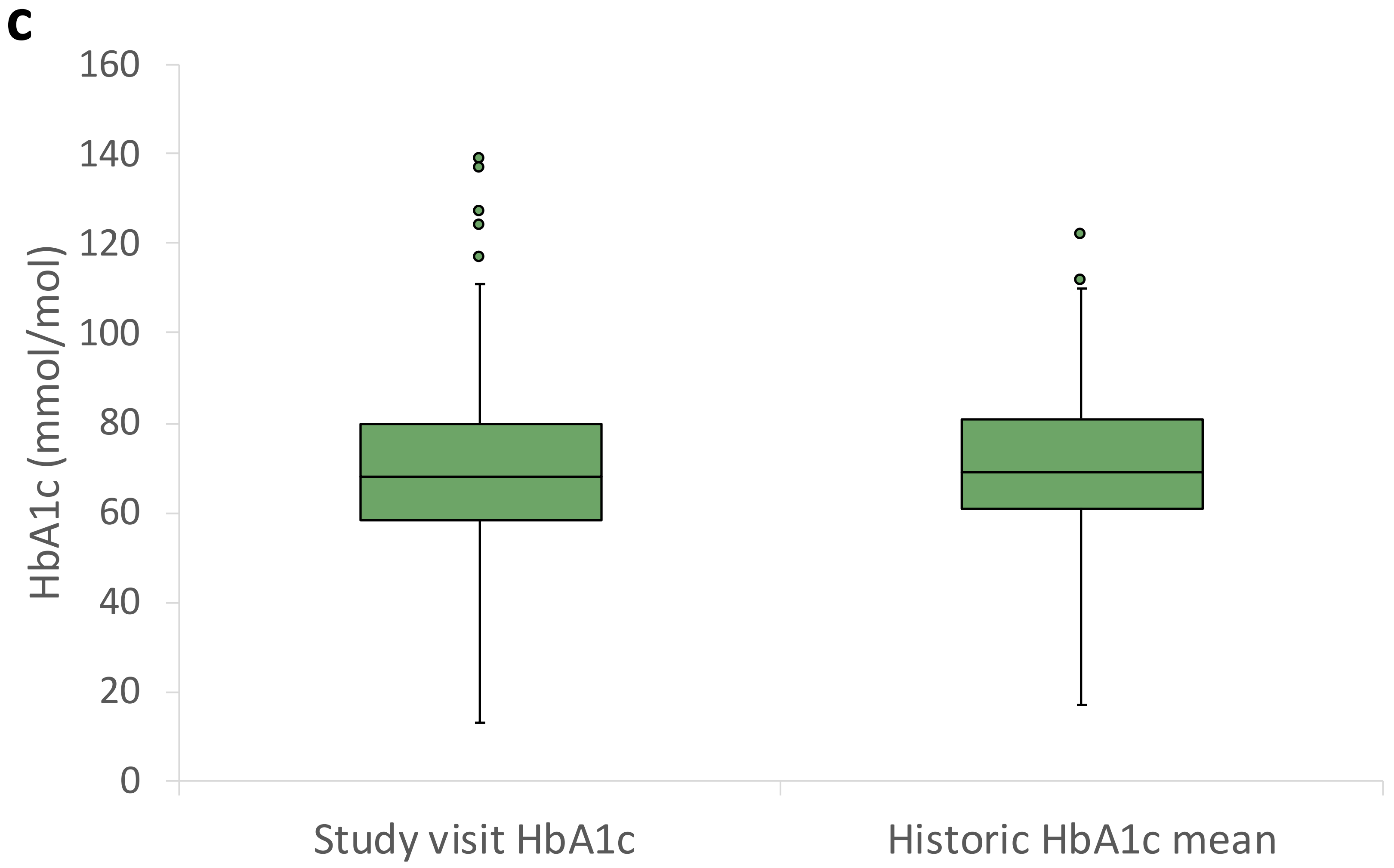

Fig. S5 – Boxplot of daily insulin dose by C-peptide group. Insulin dose was lower in the preserved C-peptide group (n=70) vs the low (n=151) group, p=0.01. Including outliers.

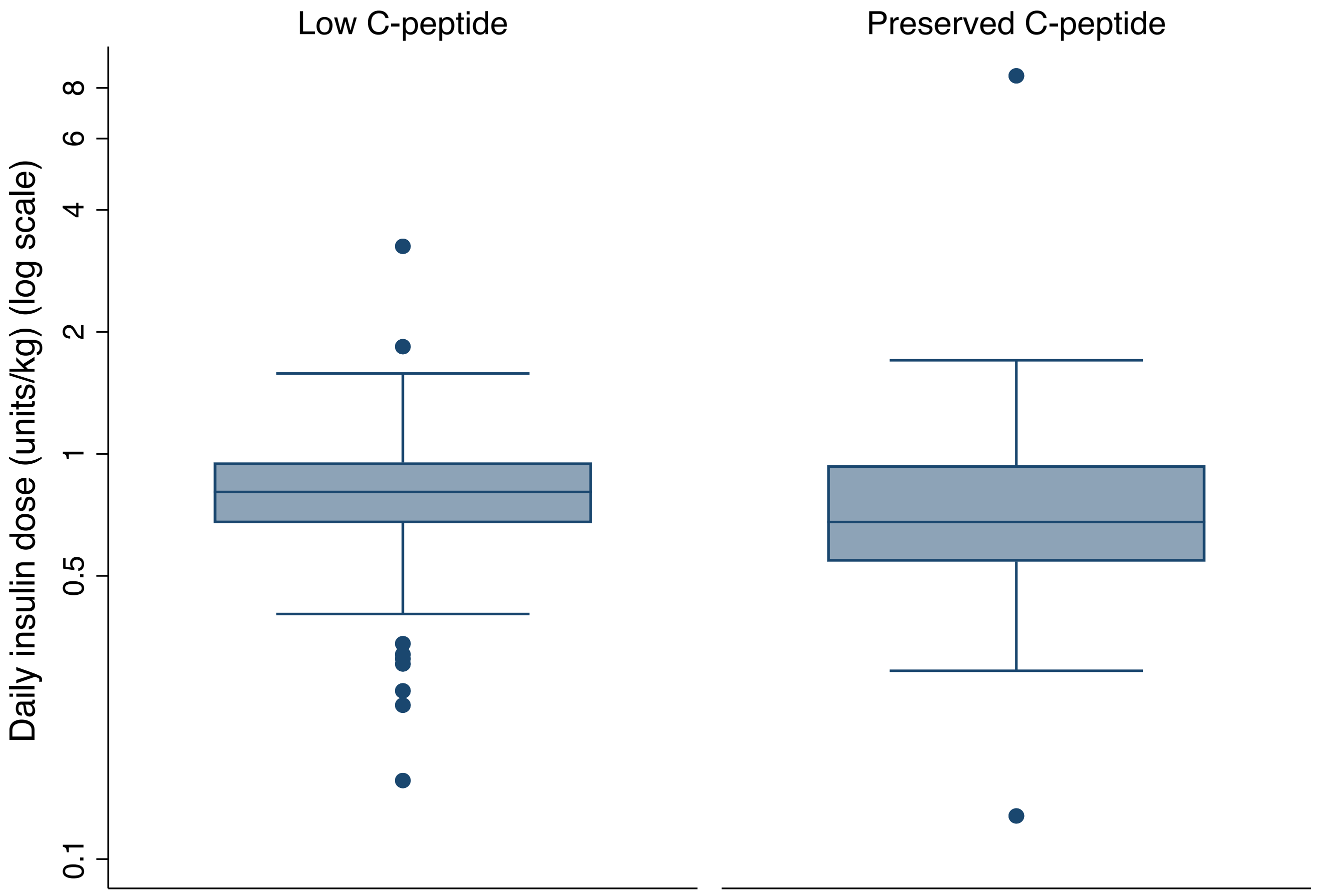

Table S2 - Complication analysis cohort characteristics. Values reported as n (%) or median (IQR). *p* values are Mann Whitney U unless otherwise stated, * denotes Chi^2.

Characteristics	Low C-peptide	Preserved C-peptide	*p* value
**No. of participants (%)**	81	59	-
**No. Male (%)**	46 (57)	23 (39)	0.04*
**Age at diagnosis (years)**	10.9 (5.2 15.2)	17.0 (12.5, 23.8)	<0.0001
**Age at recruitment (years)**	34.4 (23.2, 47.9)	34.1 (26.1, 43.6)	0.8
**BMI (kg/m²)**	24.5 (22.5, 27.2)	25.5 (22.5, 27.3)	0.6
**BMI standard deviation score**	1.0 (0.4, 1.5)	1.0 (0.3, 1.5)	0.9
**Duration of diabetes (years)**	21.3 (12.4, 37.8)	14.1 (7.8, 23.2)	0.001
**C-peptide (pmol/l)**	<3 (<3, 4)	135 (57, 314)	<0.0001
**HbA1c (mmol/mol)**	66 (58, 78)	69 (63, 81)	0.08
**HbA1c (%)**	8.2 (7.5, 9.3)	8.5 (7.9, 9.6)	0.08
**Insulin dose in 24 h (U/kg)**	0.75 (0.59, 0.88)	0.67 (0.54, 0.92)	0.2
**No. in retinopathy analysis**	74	56	-
**No. in microalbuminuria analysis**	69	51	-

Fig. S6 – Rates of retinopathy and microalbuminuria by C-peptide group. Proportion of C-peptide group with retinopathy in blue; background in dark blue, pre-proliferative in medium blue, proliferative in light blue. Proportion with retinopathy was similar in the low (n=55/74) vs preserved C-peptide group (n=37/56), p=0.3. Proportion of C-peptide group with microalbuminuria (green); similar in low (n=16/69) and preserved (n=10/51) C-peptide groups, p=0.6.

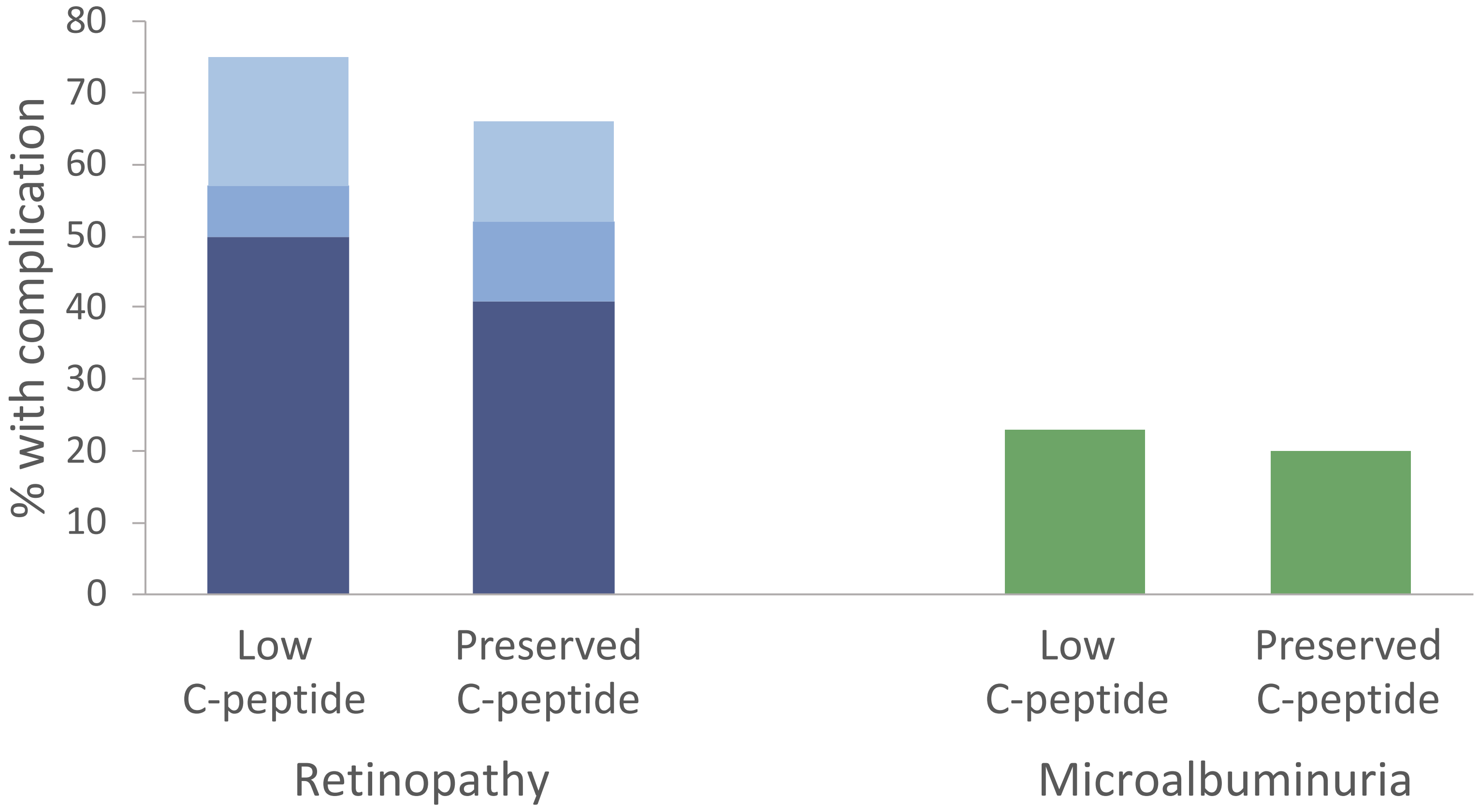

Supplement: Supplementary file 1 — Figure S1. Hypoglycaemia rates by C‐peptide group. Figure S2. Reduced hypoglycaemic awareness by C‐peptide group. Figure S3. Boxplot of study visit HbA1c by C‐peptide group. Figure S4. Historic HbA1c mean analysis. Figure S5. Boxplot of daily insulin dose by C‐peptide group. Figure S6. Rates of retinopathy and microalbuminuria by C‐peptide group. Table S1. Hypoglycaemia analysis cohort characteristics. Table S2. Complication analysis cohort characteristics. [file DME-36-1092-s001.pdf]
